# Synergistic interaction between the agonism of cebranopadol at nociceptin/orphanin FQ and classical opioid receptors in the rat spinal nerve ligation model

**DOI:** 10.1002/prp2.444

**Published:** 2018-11-28

**Authors:** Thomas Christoph, Robert Raffa, Jean De Vry, Wolfgang Schröder

**Affiliations:** ^1^ Preclinical Drug Development Grünenthal GmbH Aachen Germany; ^2^ Temple University School of Pharmacy Philadelphia Pennsylvania; ^3^ University of Arizona College of Pharmacy Tucson Arizona; ^4^ Grünenthal Innovation Grünenthal GmbH Aachen Germany; ^5^ Translational Science and Intelligence Grünenthal GmbH Aachen Germany

**Keywords:** cebranopadol, nociceptin/orphanin FQ, rat, spinal nerve ligation, synergism

## Abstract

Cebranopadol (trans‐6′‐fluoro‐4′,9′‐dihydro‐*N,N*‐dimethyl‐4‐phenyl‐spiro[cyclohexane‐1,1′(3′H)‐pyrano[3,4‐b]indol]‐4‐amine) is a novel analgesic nociceptin/orphanin FQ opioid peptide (NOP) and classical opioid receptor (MOP, DOP, and KOP) agonist with highly efficacious and potent activity in a broad range of rodent models of nociceptive, inflammatory, and neuropathic pain as well as limited opioid‐type side effects such as respiratory depression. This study was designed to explore contribution and interaction of NOP and classical opioid receptor agonist components to cebranopadol analgesia in the rat spinal nerve ligation (SNL) model. Assessing antihypersensitive activity in SNL rats intraperitoneal (IP) administration of cebranopadol resulted in ED_50_ values of 3.3 and 3.58 μg/kg in two independent experiments. Pretreatment (IP) with J‐113397 (4.64 mg/kg) a selective antagonist for the NOP receptor or naloxone (1 mg/kg), naltrindole (10 mg/kg), or nor‐BNI (10 mg/kg), selective antagonists for MOP, DOP, and KOP receptors, yielded ED_50_ values of 14.1, 16.9, 17.3, and 15 μg/kg, respectively. This 4‐5 fold rightward shift of the dose‐response curves suggested agonistic contribution of all four receptors to the analgesic activity of cebranopadol. Combined pretreatment with a mixture of the antagonists for the three classical opioid receptors resulted in an 18‐fold potency shift with an ED_50_ of 65.5 μg/kg. The concept of dose equivalence was used to calculate the expected additive effects of the parent compound for NOP and opioid receptor contribution and to compare them with the observed effects, respectively. This analysis revealed a statistically significant difference between the expected additive and the observed effects suggesting intrinsic synergistic analgesic interaction of the NOP and the classical opioid receptor components of cebranopadol. Together with the observation of limited respiratory depression in rats and humans the synergistic interaction of NOP and classical opioid receptor components in analgesia described in the current study may contribute to the favorable therapeutic index of cebranopadol observed in clinical trials.

**Abbreviations**
ANOVA: Aanalysis of variance
CFA: complete Freund's adjuvant
CI: confidence interval
DMSO: dimethyl sulfoxide
DOP: delta opioid peptide
*E*_max_: maximum possible effect for the agonist
J‐113397: 1‐[(3*R*,4*R*)‐1‐cyclooctylmethyl‐3‐hydroxymethyl‐4‐piperidyl]‐3‐ethyl‐1,3‐dihydro‐2*H*‐benzimidazol‐2‐one
KOP: kappa opioid peptide
MOP: mu opioid peptide
MPE: maximum possible effect
NOP: nociceptin/orphanin FQ opioid peptide
nor‐BNI: nor‐binaltorphimine
Ro65‐6570: 8‐acenaphthen‐1‐yl‐phenyl‐1,3,8‐triaza‐spiro[4,5]decan‐4‐one hydrochloride
SNC‐80: 4‐[(R)‐[(2S,5R)‐4‐allyl‐2,5‐dimethylpiperazin‐1‐yl](3‐methoxyphenyl)methyl]‐N,N‐diethylbenzamide
SNL: spinal nerve ligation
U‐50488H: 2‐(3,4‐dichlorophenyl)‐N‐methyl‐N‐[(1R,2R)‐2‐pyrrolidin‐1‐ylcyclohexyl]acetamide

## 1 INTRODUCTION

Cebranopadol is a first‐in‐class analgesic with agonistic activity at the nociceptin/orphanin FQ opioid peptide (NOP) receptor and the classical μ‐opioid peptide (MOP), κ‐opioid peptide (KOP), and δ‐opioid peptide (DOP) receptors.^1^, ^2^ It has subnanomolar affinity for the human and rat NOP and MOP receptors and low nanomolar affinity for the KOP and DOP receptors.^2^ After systemic administration, cebranopadol exerted highly efficacious analgesic effects in rodent models of nociceptive, inflammatory, bone cancer, and chronic mono‐ and polyneuropathic pain that were 2‐3 orders of magnitude more potent than those of morphine. Recently, we demonstrated that equianalgesic doses of cebranopadol produced less respiratory depression than fentanyl because the NOP receptor agonistic component of cebranopadol exerted a protective role by intrinsically counteracting MOP receptor‐mediated respiratory depression in rats.^3^ This finding suggests a subadditive interaction of the NOP and opioid receptor components of action of cebranopadol when it comes to this prototypic MOP receptor related side effect. On the other hand, activation of both NOP and MOP receptors contributed to antihypersensitive activity of cebranopadol in rat models of spinal nerve ligation (SNL)‐induced mono‐neuropathic pain^2^ and complete Freund's adjuvant (CFA)‐induced knee joint arthritis.^4^ Interestingly, and unlike morphine, cebranopadol was about 10‐fold more potent in rodent models of chronic neuropathic^2^, ^5^ or persistent pain^6^ as compared to other more acute pain conditions. This increase in potency in neuropathic pain models might be a result of functional NOP receptor upregulation at peripheral,^7^, ^8^, ^9^ spinal^8^, and supraspinal^10^ levels combined with synergistic interaction of activation of NOP and the classical opioid receptors, although recent data also discuss alternative contribution of the endogenous NOP system and a potential role of spinal interneurons.^11^ While agonistic activity at all four opioid receptors contribute to the in vitro profile of cebranopadol and NOP and MOP receptor‐mediated analgesic efficacies have been proven in neuropathic^2^ and inflammatory pain models^4^ in rodents, neither DOP nor KOP contributions have been assessed in vivo. Concomitant activation of NOP and MOP receptors produced additive antinociception in acute pain models in rodents^12^, ^13^ and interacted synergistically to produce antihypersensitive and antinociceptive effects in rodent models of neuropathic pain^14^ and non‐human primate models of acute pain,^15^ respectively (for review see^16^).

The concept of dose equivalence was successfully used over the last years to analyze and describe the nature of pharmacological interaction both for combinations of independent drugs and drugs featuring inherent combination of two mechanisms of action.^17^ This approach enables differentiation between subadditive, additive, and supra‐additive interaction comparing experimental potency and efficacy with the theoretically additive interaction of two independent drugs or mechanisms. Numerous examples are published supporting the value of this concept in preclinical models of experimental pain.^18^, ^19^, ^20^, ^21^, ^22^

The application of this concept to a compound like cebranopadol targeting all four opioid receptors crucially depends on carefully controlled experimental conditions. Recently, in the rat SNL model with mechanical readout, we analyzed the interaction of opioid receptor agonists and antagonists for efficacy and selectivity.^23^ Fully efficacious doses of the prototypic receptor agonists Ro65‐6570 (NOP), morphine (MOP), SNC‐80 (DOP), and U‐50488H (KOP) were combined with several doses of the four antagonists J‐113397 (NOP), naloxone (MOP), naltrindole (DOP), and nor‐BNI (KOP). This data set allowed us to select selective and specific antagonistic doses to be used in the present study.

The aim of the present study was to further characterize the mode of action of cebranopadol in SNL rats by exploring the role of DOP and KOP receptors and to elucidate the way activation of NOP and classical opioid receptors interact to produce antihypersensitivity.

## 2 MATERIALS AND METHODS

### 2.1 Animals

Two hundred and twenty‐eight male Sprague‐Dawley rats were used (body weight 140‐160 g; Janvier Labs, Le Genest Saint Isle, France). Animals were housed under standard conditions (room temperature 20°C‐24°C, 12 hour light−dark cycle, relative air humidity 35%‐70%, 10‐15 air changes per hour, air movement <0.2 m/sec) with food and water available ad libitum in the home cage. Animals were assigned randomly to treatment groups. Ten rats were used per group. Different doses and vehicles were tested in a randomized fashion. Animals were tested repeatedly with a washout period of at least 1 week between tests. Although the operators performing the behavioral tests were not formally “blinded” with respect to the treatment, they were not aware of the study hypothesis or the nature of differences between drugs.

Animal testing was performed in accordance with the recommendations and policies of the International Association for the Study of Pain^24^ and the German Animal Welfare Law. All study protocols were approved by the local government authority for animal research, which are advised by an independent Ethics Committee.

### 2.2 Spinal nerve ligation

#### 2.2.1 Experimental preparation

Under pentobarbital anesthesia (Narcoren^®^ 60 m/kg IP; Merial GmbH, Hallbergmoos, Germany), the L5/L6 spinal nerves were tightly ligated according to the method by Kim and Chung.^25^ The left L5 and L6 spinal nerves were exposed by removing a small piece of the paravertebral muscle and a part of the left spinous process of the L5 lumbar vertebra. The L5 and L6 spinal nerves were then carefully isolated and tightly ligated with silk (NC‐silk black, USP 5/0, metric 1, Braun Melsungen AG, Melsungen, Germany). After checking hemostasis, the muscle and the adjacent fascia were closed with sutures and the skin was closed with sutures. After surgery, animals were allowed to recover for 1 week.

#### 2.2.2 Antihypersensitive testing

Animals developed tactile hypersensitivity which was stable for at least 5 weeks. For the assessment of tactile hypersensitivity, rats were placed on a metal mesh covered with a plastic dome and were allowed to habituate until the exploratory behavior diminished. Threshold for tactile hypersensitivity was measured with an electronic von Frey anesthesiometer (Somedic, Malmö, Sweden). Animals were tested 30 minutes prior to intraperitoneal (IP) administration of cebranopadol or vehicle and 20, 50, and 80 minutes after IP administration of cebranopadol or vehicle. The median withdrawal threshold for each animal at a given time was calculated from five individual stimulations with the electronic von Frey filament. Withdrawal thresholds of the injured paws are expressed as percent of the maximum possible effect (MPE) by comparing predrug threshold of SNL animals (=0% MPE) and control threshold of sham animals (=100% MPE). A cutoff was set at 100% MPE: values above 100% were considered as 100%. The effect of cebranopadol and vehicle was calculated for each testing time point as interindividual %MPE value. In antagonism experiments, J‐113397 4.64 mg/kg IP (Grünenthal GmbH, Aachen, Germany), naloxone 1 mg/kg IP (Sigma, Taufkirchen, Germany), naltrindole 10 mg/kg IP (Tocris, Bristol, UK), nor‐binaltorphimine 10 mg/kg IP (Biotrend, Cologne, Germany), or vehicle (0.9% NaCl) was administered 10 minutes before cebranopadol or vehicle (10% DMSO/5% Cremophor EL/85% glucose solution (5%)).

### 2.3 Data analysis

Data were analyzed by means of two‐factor analysis of variance (ANOVA), with repeated measures. Significance of treatment, time, or treatment by time interaction effects was analyzed by means of Wilks’ Lambda. In case of a significant treatment effect, pairwise comparisons were performed by post hoc analysis using the Bonferroni test. Results were considered statistically significant if *P *<* *0.05. ED_50_ values and 95% confidence intervals (CIs) were determined at the time of the peak effect by linear regression analysis based on %MPE data.

### 2.4 Analysis of interaction between NOP and opioid receptor agonistic components of cebranopadol

The concept of dose equivalence^17^ was used to analyze the interaction between the NOP receptor component (combined MOP/DOP/KOP receptor antagonism by triple combination of naloxone, naltrindole, and nor‐BNI) and the opioid receptor component (NOP receptor antagonism by J‐113397) of cebranopadol. Based on dose‐effect (*D‐E*) curves (log dose) the expected effect can be described as *E = E*_max_ *D*/(*D* +* C*)*,* where *E*_max_ is the maximum effect and *C* is the constant that describes the drug's potency. In the current analysis, *C* presents the doses for the respective half‐maximal effects (ED_50_) of cebranopadol after pretreatment with the opioid receptor antagonists or the NOP receptor antagonist. First, dose equivalents (DE) were calculated for both the NOP and opioid receptor components (Table 1). Second, for each dose of cebranopadol, the paired expected (additive) effects associated with the effect *E*_NOP_ mediated by NOP receptor agonism, and the effect *E*_opioid_ mediated by opioid receptor agonism were calculated according to the following equations:

$$E_{\mathrm{NOP}}=\frac{100\;{\text{DE}}_{\mathrm{NOP}}}{{\mathrm{DE}}_{\mathrm{NOP}}+65.5}\;\text{and}\;E_{\mathrm{Opioid}}=\frac{100\;{\text{DE}}_{\mathrm{Opioid}}}{{\text{DE}}_{\mathrm{Opioid}}+14.1}$$

**Table 1:** Calculation of NOP and opioid dose equivalents (DE). D represents the respective dose of cebranopadol

Cebranopadol dose (μg/kg)	Dose equivalent	Dose equivalent
	NOP (μg/kg)	Opioid (μg/kg)
	${\text{DE}}_{\mathrm{NOP}}=D+D\frac{C_{\mathrm{NOP}}}{C_{\mathrm{Opioid}}}$	${\text{DE}}_{\mathrm{Opioid}}=D+\frac{D}{C_{\mathrm{NOP}}/C_{\mathrm{Opioid}}}$
0.8	4.5	1.0
1.72	9.7	2.1
3.71	21.0	4.5
8	45.2	9.7
17.2	97.2	20.9
37.1	209.6	45.1
80	452.0	97.2
172	971.8	209.0
252.8	1428.3	307.2

For each dose of cebranopadol, the calculated expected (additive) effect was compared with the observed effect (Table 2). The resulting data were analyzed by Student's *t* test for paired data (*E*_additive_ vs *E*_observed_).

**Table 2:** Comparison of observed and calculated antiallodynic effects of cebranopadol

Cebranopadol	Observed effect		Calculated effect	Test for synergy	Observed effect		Calculated effect	Test for synergy
	*E*_NOP_	Control	*E*_NOP_	*E*_NOP_	*E*_opioid_	Control	*E*_opioid_	*E*_opioid_
	Triple opioid receptor antagonism	Vehicle pretreatment	$E_{\mathrm{NOP}}=\frac{100{\text{DE}}_{\mathrm{NOP}}}{{\text{DE}}_{\mathrm{NOP}}+C_{\mathrm{NOP}}}$		NOP receptor antagonism	Vehicle pretreatment	$E_{\mathrm{Opioid}}=\frac{100{\text{DE}}_{\mathrm{Opioid}}}{{\text{DE}}_{\mathrm{Opioid}}+C_{\mathrm{Opioid}}}$	
Dose [μg/kg]	MPE [%]	MPE [%]	MPE [%]	*d*	MPE [%]	MPE [%]	MPE [%]	*d*
0.8		16.6	6.5	−10.1		6.1	6.5	0.4
1.72		33.5	13.0	−20.5		38.3	13.0	−25.3
3.71		50.3	24.3	−26.0		56.5	24.3	−32.3
8		59.4	41.0	−18.4	27.4	71.5	41.0	−30.5
17.2	12.4	94.4	59.9	−34.5	61.7	93.6	59.9	−33.7
37.1	31.3		76.3		85.1		76.3	
80	50.7		87.4		98.5		87.4	
172	79.3		93.7				93.7	
252.8	94.9		95.6				95.6	
ED_50_ [μg/kg]	*C*_NOP_: 65.5	3.58			*C*_Opioid_: 14.1	3.3		

Calculated effects reflect a supposedly additive interaction of the NOP and opioid components of action. DE represents the respective dose equivalent as calculated in Table 1. *d* represents the difference between observed and calculated effects.

### 2.5 Drugs and chemicals

The following drugs were used: cebranopadol hemi‐citrate (Grünenthal GmbH, Aachen, Germany), J‐113397 (*CAS no.: 2177461‐40‐0;* Grünenthal GmbH, Aachen, Germany), sodium pentobarbital (*CAS no.: 57‐33‐0;* Narcoren^®^), naloxone HCl (*CAS no.: 51481‐60‐8;* Sigma‐Aldrich Chemie GmbH, Taufkirchen, Germany), naltrindole (*CAS no.: 111469‐81‐9;* Tocris, Bristol, UK), and nor‐BNI dihydrochloride (*CAS no.: 105618‐26‐6;* Biotrend, Cologne, Germany).

The following chemicals were used: cremophor EL, DMSO, 5% glucose (Sigma‐Aldrich Co., St Louis, MO, USA; Sigma‐Aldrich Chemie GmbH, Munich, Germany), and physiological saline (0.9% NaCl, Baxter, Cherry Hill, NJ, USA; Baxter, Unterschleißheim, Germany).

Cebranopadol hemi‐citrate was dissolved in 10% DMSO/5% Cremophor EL/85% glucose solution (5%). J‐113397, naloxone, naltrindole, and nor‐BNI were dissolved in 0.9% NaCl. Administration volume was 5 mL/kg.

Cebranopadol was tested as the hemi‐citrate salt. All doses and ED_50_ values refer to the respective free base. For simplicity, the salt forms have been omitted from the text.

## 3 RESULTS

### 3.1 Dose‐dependent antihypersensitivity

**Figure 1:**
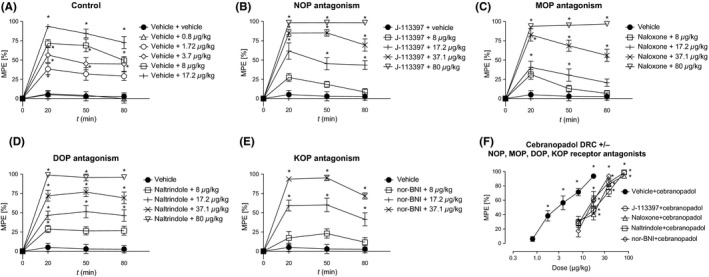
Dose‐ and time‐dependent antiallodynic effect of intraperitoneal cebranopadol after IP pretreatment with vehicle (A), J‐113397 (4.64 mg/kg, (B)), naloxone (1 mg/kg, (C)), naltrindole (10 mg/kg, (D)), and nor‐BNI (10 mg/kg, (E)). Dose‐response curves of cebranopadol after pretreatment with vehicle or antagonists 20 minutes after agonist administration (F). *P < 0.05 vs vehicle

In the first set of experiments, after pretreatment with vehicle, cebranopadol was tested at doses of 0.8, 1.72, 3.71, 8 and 17.2 μg/kg IP and produced dose‐ and time‐dependent inhibition of mechanical hypersensitivity (treatment: *F*_5,54_ = 35.077, *P* < 0.0001; time: *F*_2,108_ = 12.481, *P* < 0.0001; interaction: *F*_10,108_ = 1.298, *P* = 0.241; Figure 1A and F). The highest dose tested showed full efficacy with 94% MPE. Potency was quantified by an ED_50_ value (95% CI) of 3.3 (2.66‐4.04) μg/kg IP, calculated from the peak effect vs control values at 20 minutes after administration.

### 3.2 Antagonism of antihypersensitivity by NOP and opioid receptor antagonists

The antagonist doses used in this study were previously demonstrated to completely and selectively inhibit full antiallodynic efficacy of NOP and opioid receptor selective agonists in the rat SNL model.^23^

Pretreatment with the selective NOP receptor antagonist J113397 (4.64 mg/kg IP) resulted in a 4.3‐fold rightward shift of the dose‐dependent antiallodynic effect of cebranopadol (8‐80 μg/kg IP; treatment: *F*_5,54_ = 101.418, *P* < 0.0001; time: *F*_2,108_ = 6.344, *P* = 0.002; interaction: *F*_10,108_ = 1.611, *P* = 0.113) with maximum efficacy of 99% MPE and an ED_50_ (95% CI) of 14.1 (10.3‐17.7) μg/kg IP, 20 minutes after agonist treatment (Figure 1B and F).

Pretreatment with the selective MOP receptor antagonist naloxone (1 mg/kg IP) resulted in a 5.1‐fold rightward shift of the dose‐dependent antiallodynic effect of cebranopadol (8‐80 μg/kg IP; treatment: *F*_5,54_ = 64.306, *P* < 0.0001; time: *F*_2,108_ = 14.929, *P* < 0.0001; interaction: *F*_10,108_ = 3.172, *P* = 0.001) with maximum efficacy of 97% MPE and an ED_50_ (95% CI) of 16.9 (12.5‐21.4) μg/kg IP, 20 minutes after agonist treatment (Figure 1C and F).

Pretreatment with the selective DOP receptor antagonist naltrindole (10 mg/kg IP) resulted in a 5.2‐fold rightward shift of the dose‐dependent antiallodynic effect of cebranopadol (8‐80 μg/kg IP; treatment: *F*_5,54_ = 72.351, *P* < 0.0001; time: *F*_2,108_ = 0.413, *P* = 0.663; interaction: *F*_10,108_ = 0.284, *P* = 0.983) with maximum efficacy of 99% MPE and an ED_50_ (95% CI) of 17.3 (14.2‐20.5) μg/kg IP, 20 minutes after agonist treatment (Figure 1D and F).

Pretreatment with the selective KOP receptor antagonist nor‐BNI (10 mg/kg IP) resulted in a 4.5‐fold rightward shift of the dose‐dependent antiallodynic effect of cebranopadol (8‐37.1 μg/kg IP; treatment: *F*_4,45_ = 52.318, *P* < 0.0001; time: *F*_2,90_ = 8.279, *P* = 0.001; interaction: *F*_8,90_ = 2.405, *P* = 0.021) with maximum efficacy of 95% MPE and an ED_50_ (95% CI) of 15 (12.7‐17.5) μg/kg IP, 20 minutes after agonist treatment (Figure 1E and F).

### 3.3 Synergistic interaction between NOP and opioid receptor agonistic components of cebranopadol

**Figure 2:**
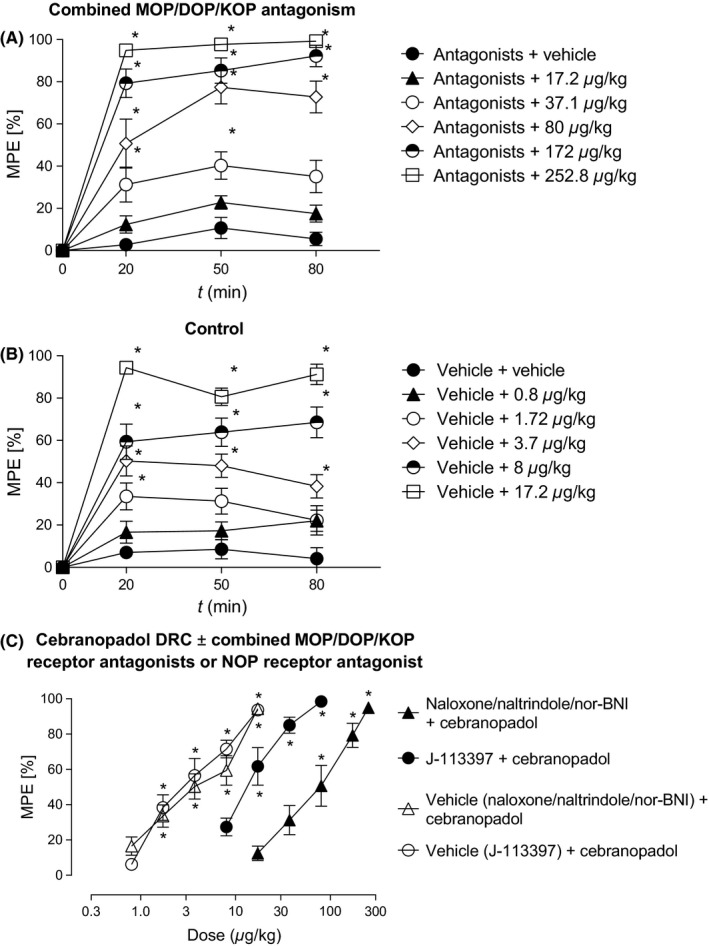
Dose‐ and time‐dependent antiallodynic effect of intraperitoneal cebranopadol after IP pretreatment with a triple combination of opioid receptor antagonists (naloxone 1 mg/kg, naltrindole 10 mg/kg, and nor‐BNI 10 mg/kg, (A)) and the corresponding vehicle control (B). Dose‐response curves of cebranopadol after pretreatment with vehicle or antagonists 20 minutes after agonist administration (C). *P < 0.05 vs vehicle

The two components of action (ie, NOP receptor agonism and classical opioid receptor agonism) are a feature of the parent compound. In experimental settings, these two components can be viewed the same way as that one would deal with two different drugs. Thus, the concept of dose equivalence, which is also the basis of isobolographic analysis, could be used to analyze the nature of interaction (ie, additive, synergistic, subadditive) between the NOP receptor agonistic and the opioid receptor agonistic component of cebranopadol to produce antiallodynic efficacy. We based our analysis on a comparison of observed and expected (additive) effect scales of cebranopadol according to 17. To this end, the dose‐effect relations of the two individual components had to be obtained by selective antagonism. The NOP receptor agonistic component of cebranopadol was isolated in a second set of experiments by pretreatment with a triple combination of the MOP, DOP, and KOP receptor antagonists naloxone (1 mg/kg IP), naltrindole (10 mg/kg IP), and nor‐BNI (10 mg/kg IP), respectively that resulted in a 18.3‐fold rightward shift of the dose‐dependent antiallodynic effect of cebranopadol (17.2‐52.8 μg/kg IP; treatment: *F*_5,54_ = 70.459, *P* < 0.0001; time: *F*_2,108_ = 6.258, *P* = 0.003; interaction: *F*_10,108_ = 1.299, *P* = 0.24) yielding maximum efficacy of 99% MPE and an ED_50_ (*C*_NOP_) (95% CI) of 65.5 (52.3‐81.1) μg/kg IP, 20 minutes after agonist treatment (Fig. 2A and C). The corresponding vehicle control resulted in dose‐dependent inhibition of hypersensitivity (treatment: *F*_5,54_ = 48.350, *P* < 0.0001; time: *F*_2,108_ = 0.532, *P* = 0.589; interaction: *F*_10,108_ = 1.607, *P* = 0.114) with a maximum efficacy of 94% MPE and an ED_50_ (95% CI) of 3.58 (2.79‐4.57) μg/kg IP, 20 minutes after agonist treatment (Figure 2B and C). The MOP, DOP, and KOP receptor‐mediated opioid agonistic component of cebranopadol had been isolated by pretreatment with the selective NOP receptor antagonist J113397 that yielded maximum efficacy of 99% MPE and an ED_50_ (*C*_opioid_) (95% CI) of 14.1 (10.3‐17.7) μg/kg IP as shown in the first set of experiments above (Figures 1B and F and 2C).

As maximal efficacies were identical (*E*_max_ = 99% MPE), the regression lines of the dose‐effect relations for NOP receptor and opioid receptor‐mediated components of action were tested for parallelism (Figures 2C). Using the root mean square error value and degree of freedom for each regression line, *s*_*p*_ = {[(3)(4.28)^2^ + (2)(7.42)^2^]/5}^&1/2;^ = 5.75, from which *t *= (30.57‐30.83)/[5.75(1/54.89 + 1/110.20)^&1/2;^] = −0.280. Because −0.280 is smaller than the *t*_Table_ value (*P* = 0.05; df = 5) of 2.571, the two lines are not significantly different from parallel. Since the regression lines of the dose‐effect relations were parallel, a potency ratio for NOP and opioid receptor agonism, ED_50_(NOP)/ED_50_(Opioid), of 65.5/14.1 = 4.65 could be derived that is assumed to be constant over the whole range of dose‐effect curves. The concept of dose equivalence was now used to calculate the expected effect of cebranopadol that would arise from additive contributions of its two components of action.

A comparison of the observed (experimental) and calculated (additive) effects is given in Table 2. Statistical testing was performed by means of a two‐sided Student's *t* test for paired data according to the procedure described in 17. For the NOP receptor component, the vehicle data were tested against the corresponding expected (additive) NOP data and revealed a statistically significant difference (Student *t* test for paired data (*E*_additive_ vs *E*_observed_); df = 4; *t* = −3.84; *P* = 0.0184). For the opioid receptor component, the vehicle data were tested against the corresponding expected (additive) opioid data and resulted in a statistically significant difference (Student *t* test for paired data (*E*_additive_ vs *E*_observed_); df = 4; *t* = −5.40; *P* = 0.0057). Thus, the results of this analysis showed that the observed effect magnitude at each tested dose of cebranopadol exceeded the calculated (additive) effect of its NOP and classical opioid component of action, a finding that indicates a synergistic interaction between the two distinct modes of action of cebranopadol.

## 4 DISCUSSION

The novel centrally acting analgesic cebranopadol is a first‐in‐class potent NOP and opioid receptor agonist that displayed broad analgesic activity in preclinical models of acute, inflammatory, and chronic neuropathic pain and is currently under clinical development for the treatment of severe chronic nociceptive and neuropathic pain.^1^, ^2^

Previously, we showed that intravenous administration of cebranopadol exerted potent and fully efficacious antiallodynic activity that was dose‐dependently inhibited by the NOP receptor antagonist J‐113397 as well as the MOP receptor antagonist naloxone in the rat SNL model of mono‐neuropathic pain.^2^ The present study corroborates and extends these earlier findings by demonstrating that activation of NOP, MOP, DOP, and KOP receptors equally contributed to antihypersensitive activity in SNL rats as the respective selective receptor antagonists J‐113397, naloxone, naltrindole, and nor‐BNI caused nearly identical rightward shifts of the dose‐response curve of intraperitoneal cebranopadol. Previously, we demonstrated that the doses of the antagonists used in this study showed sufficient selectivity and efficacy in the rat SNL model to characterize relative antihypersensitive contributions of all four receptors.^23^ Importantly, a comparison of observed and expected effect scales that was calculated based on the concept of dose equivalence^17^ revealed that NOP receptor activation interacted synergistically with activation of classical opioid (MOP, DOP, and KOP) receptors to produce antiallodynic efficacy of systemic cebranopadol in this rodent model of chronic neuropathic pain.

Several behavioral pharmacology studies reported on interactions between NOP and classical opioid receptors in rodent models of neuropathic pain. For example, in addition to NOP receptor activation also spinal MOP, DOP, and KOP receptors contributed to antiallodynic efficacy of spinal N/OFQ in SNL rats though N/OFQ is unable to bind and activate classical opioid receptors.^26^ Moreover, investigating receptor subtype‐selective agonists in corresponding NOP and classical opioid receptor knockout mice revealed complex interactions between NOP and classical opioid receptors in a mouse model of diabetic polyneuropathic pain, in particular with NOP receptors being functionally interlinked to DOP and KOP receptors.^27^ Notably, by applying isobolographic analysis, one study investigated the mode of interaction of the spinally administered NOP and MOP receptor agonists N/OFQ and morphine in the rat chronic constriction injury model of mono‐neuropathic pain and demonstrated synergistic inhibition of mechanical hyperalgesia.^14^ The studies using combinations of spinally administered agonists and antagonists delineated the spinal cord as one site of (synergistic) interaction, whereas the pharmacogenomics study based on global NOP and opioid receptor knockout did not allow drawing any conclusion on the anatomical substrate(s) where the complex NOP‐opioid receptor interaction occurred. Likewise, we cannot ascribe the precise site(s) of synergistic interaction between cebranopadol's NOP and classical opioid receptor agonistic mechanisms of action as both cebranopadol and antagonists were administered systemically in the present study. Although cebranopadol was demonstrated to produce antihypersensitive efficacy after peripheral, spinal, and supraspinal administration in rodent models of chronic neuropathic pain, the site‐specific relative contribution and way of interaction between NOP and classical opioid receptor agonistic MoA still remains elusive as no antagonism experiments were conducted in the context of that study.^28^ In addition, also site‐site interactions might contribute to produce NOP and opioid receptor synergism of cebranopadol as has been described for the MOR‐NRI mediated intrinsic synergism of tapentadol.^19^ Intrinsic synergism of a compound such as cebranopadol when it would be based on interaction of NOP and opioid agonistic efficacy at multiple sites relevant to pain processing requires equal distribution throughout the different compartments within the body. In fact, the pharmacokinetic profile of cebranopadol in rats suggests rapid absorption and extensive distribution^2^ enabling equal NOP and opioid receptor activation at potential sites of synergism such as the spinal cord.

The complexity of local and site‐site activation of NOP and classical opioid receptors might well lay the ground to the analgesic synergism detected in the current study. Furthermore, the nature of molecular receptor activation might contribute to the beneficial therapeutic index of cebranopadol. In fact, functional studies revealed a G protein biased signaling of cebranopadol at the NOP and at a reduced degree at the MOP receptor.^6^ Reduced ß‐arrestin recruitment and preferred G protein activation are discussed as contributor for reduction of opioid‐type side effects such as respiratory depression and gastrointestinal dysfunction.^29^

Respiratory depression is a clinical issue of pure MOP receptor agonists like morphine and fentanyl.^30^ Thus, an obvious question based on the present finding is whether synergistic interaction between NOP and classical opioid receptor agonists is also reflected in an increase in opioid‐type side effects. Notably, cebranopadol was largely devoid of a respiratory depressant effect in the clinic.^31^ In a preclinical model in rats, the NOP receptor agonistic component of cebranopadol was demonstrated to counteract MOP receptor‐mediated respiratory depression.^3^

Impairment of motor coordination is another opioid‐type side effect targeting the central nervous system in rodents. Similar to the situation in the respiratory system, cebranopadol does not show efficacy in the rotarod test at doses which exceed antinociceptive or antihypersensitive doses in rats^2^ or mice.^6^ This finding suggests lack of confounding motor effects in behavioral assays increasing the confidence in the current data set. More importantly, this data corroborates the finding on respiration, that is lack of synergism in opioid‐type side effects as compared to synergistic interaction in analgesia.

The scope of the current study was to elucidate the interaction of NOP and classical opioid receptor agonism for cebranopadol. The in vitro binding profile shows predominant binding to NOP and MOP and weaker affinity to DOP and KOP,^2^ which is also reflected in functional efficacies.^2^, ^6^ Hence, we first analyzed the functional contribution of all four receptors in vivo before we assessed the interaction of NOP and classical opioid receptors by the concept of dose equivalence. Interestingly, when using isolated antagonists the shift of the dose‐response curves was similar in magnitude for all four receptors despite differential affinities and potencies in vitro. The data suggest a complex interaction between the different opioid receptors which also is reflected in the outcome of genetic models in mice^27^ and antagonism studies in rats.^23^ Further dissection of this complex opioid receptor interaction might be possible in a similar experimental setup in vivo but was out of the scope of the current study and would require considerably higher numbers of animals contradicting the 3Rs principles of animal welfare.

Thus, NOP receptor agonism of cebranopadol both afforded intrinsic limitation of MOP receptor‐mediated respiratory depression and motor impairment and contributed synergistically to opioid receptor‐mediated antiallodynic efficacy. This two pronged beneficial effect of the NOP receptor agonistic component is therefore believed to contribute to the favorable therapeutic index of cebranopadol in the clinic.^32^, ^33^

## AUTHORS’ CONTRIBUTIONS

*Participated in research design:* Christoph, De Vry.

*Conducted experiments:* Christoph.

*Performed data analysis:* Christoph, Raffa, Schröder.

*Wrote or contributed to the writing of the manuscript:* Christoph, Raffa, Schröder.
